# Awareness of the 2009 US Preventive Services Task Force recommended changes in mammography screening guidelines, accuracy of awareness, sources of knowledge about recommendations, and attitudes about updated screening guidelines in women ages 40–49 and 50+

**DOI:** 10.1186/1471-2458-12-899

**Published:** 2012-10-24

**Authors:** Marc T Kiviniemi, Jennifer L Hay

**Affiliations:** 1 Department of Community Health and Health Behavior, University at Buffalo, 314 Kimball Tower, 3435 Main Street, Buffalo, NY, 14222, USA; 2 Department of Psychiatry and Behavioral Sciences, Memorial Sloan-Kettering Cancer Center, 330 E 59th St, 7th floor, New York, NY, 10022, USA

**Keywords:** Screening recommendations, Mammography, Health communication, Attitudes about public health recommendations, Awareness of changes

## Abstract

**Background:**

The US Preventive Services Task Force updated mammography recommendations in 2009, recommending against routine screening for women ages 40–49 and reducing recommended frequency for women 50+. The recommendation changes were highly controversial and created conflicting recommendations across professional organizations. This study examines overall awareness of the changes, accuracy of knowledge about changes, factors related to both overall awareness and accuracy, sources of knowledge about changes, and attitudes about the new recommendations.

**Method:**

National telephone survey of 508 women, half aged 40–49 and half 50+, conducted one year after the update (November/December 2010; cooperation rate was 36%). Measures include awareness, accuracy, source of knowledge, interactions with providers, and attitudes about the changes.

**Results:**

Fewer than half of women were aware of the guideline changes. Younger, more educated, and higher income women were more aware. Of those who were aware, only 12% correctly reported both change in age and frequency. Accuracy was not associated with demographics. The majority learned of changes through the media and the majority had negative attitudes about the changes.

**Conclusions:**

Despite widespread coverage of the recommendation changes, overall awareness in the relevant population is low. Increasing awareness and addressing attitudes about the changes is necessary to ensure the use of recommendations to impact screening behavior.

## Background

Public health organizations frequently release guidelines for recommended behavioral practices to advance public health goals. These guidelines cover a range of behavioral areas of public health relevance, including vaccination, dietary behavior, substance use, and disease detection screening. The effectiveness of such messages depends on public awareness of the recommendations and on how the public responds to the behavioral recommendations. However, awareness of and reactions to recommendations are often not assessed.

In November 2009, the United States Preventive Services Task Force (USPSTF) issued revised recommendations for breast cancer screening mammography for average risk women [1]. From 2002–2009, USPSTF guidelines recommended routine screening at least every two years for women aged 40–49 and annual mammography for women aged 50 and older [2,3]. The 2009 update recommended against routine screening for women 40–49, stating that “…the decision to start regular, biennial screening mammography before the age of 50 years should be an individual one and take patient context into account, including the patient's values regarding specific benefits and harms” [1]; for women aged 50–74 the Task Force reduced the recommended frequency from annual screening to screening every other year.

The release of the revised recommendations was widely reported in the mass media and became a contentious discussion point in the debate around health care reform [4]. The changes were also widely debated in professional circles [4,5]. Other professional organizations did not change recommendations; for example, the American Cancer Society and the Susan G. Komen Foundation continued to recommend annual screening beginning at age 40 [6,7]. In some cases, professional organizations actively and publicly expressed disagreement with the USPSTF recommendation changes [8], and the differences in recommendations across organizations was highlighted in some of the media commentary on the issue [9].

Similar professional debates and media attention have surrounded previous changes in mammography screening recommendations [10,11]. Although a great deal of media attention was given to the changes in recommendations and a substantial public debate ensued, little is known about the degree of awareness of, knowledge about, and attitudes about the changes among women in the relevant age groups for the recommended changes (women age 40–49 and women ages 50 and older).

Mammography has been described as one of the important “success stories” of cancer screening, with reductions in breast cancer mortality attributed in part to mammography utilization [12]. Rates of screening adherence are high for mammography relative to other cancer screening modalities [13] (self-reported screening rates in the US were over 70% from 2000–2007 [14]). Given these facts, understanding factors that relate to awareness of the changes to the USPSTF mammography screening recommendations, accuracy of knowledge about the changes and attitudes about the changes is critical for anticipating the public health impact of these updated recommendations.

Examination of previous screening controversies has shown that public awareness is often low [15,16]. A number of factors may be relevant to understanding awareness of changes. First, demographic and socioeconomic factors are broadly associated with screening compliance [17,18] and might therefore be associated with awareness, as well [15]; one would predict lower awareness in populations associated with lower health literacy and greater knowledge gaps (e.g., low SES populations). For breast cancer, age is of specific interest because many of the recommendation changes in breast cancer screening over time, including those made by the USPSTF in 2009, involve changes in age of screening; one would predict greater awareness for those who are directly impacted by the changes (in this case, women 40–49). In addition, familial history of breast cancer is associated with risk perception and interest in preventive actions [19-21] and may therefore influence reactions to recommendations.

This study examined awareness of and accuracy of knowledge about breast cancer screening recommendations made by the USPSTF for United States women aged 40–49, and 50 and over. Overall awareness of changes to recommendations is an important marker of the degree to which public health recommendations are disseminated to the public, including issues of whether effective communications channels are being used to announce changes. Over and above overall awareness, the degree to which those who are aware have accurate knowledge of recommendations is also important. Given that recommendations are intended as a guide to action, accurate knowledge of those changes is a necessary precursor to following the recommendations.

Data was collected one year after the USPSTF recommended changes were released to the public (the release processes and publicity surrounding the recommendation changes is described in detail elsewhere [22]). We examined levels of awareness and knowledge of adjusted recommendations, as well as potentially important factors that relate to degree of awareness and knowledge of recommendations. Finally, given that attitudes about behaviors are an important precursor to behavioral engagement [23], we examined women’s attitude about the recommendations. We surveyed equal numbers of those women older and younger than age 50, given that awareness, and factors related to awareness, of the new recommendations may differ in these age cohorts.

## Methods

### Participant recruitment and survey delivery

The survey study was conducted in November and December, 2010, approximately 1 year after the release of the updated guidelines (guidelines were published in the November 17, 2009 issue of the *Annals of Internal Medicine*[1])*.* The study protocol was approved by the Social and Behavioral Sciences IRB at the University at Buffalo.

Women ages 40 and above were recruited for the survey. Recruiting was stratified so that approximately half of the sample was women ages 40–49 and the other half was women ages 50+. A commercially available targeted sampling frame was used to select United States phone numbers likely to include at least one household member who was a woman age 40 or older. A pre-notification letter was sent to households included in the sample. Numbers in the sampling frame were called to recruit participants. Up to 9 contact attempts were made for each telephone number and a conversion attempt was made for individuals who initially declined to participate. Women who were eligible and agreed to participate immediately completed the survey by telephone.

The initial sampling frame contained 4000 potential household telephone numbers. After the study began, an additional 800 telephone numbers were added to the sampling frame (these additional numbers did not receive a pre-notification letter). Ultimately, contact attempts were made for 4671 of the 4800 numbers in the frame. Of these 4671 households, 1444 were excluded because they were not valid household numbers (e.g., FAX number, business number). An additional 1737 were excluded because they were not able to be contacted. Of the 2436 households that were successfully contacted, 1027 were excluded because there was no female age 40+ in the household or because the female age 40+ had a previous cancer diagnosis.

These exclusions led to 1409 households with an eligible respondent. Of those, 508 respondents agreed to participate and completed the interview and 901 declined to participate. Based on the American Association for Public Opinion Research’s guidelines [24], the resultant cooperation rate for the survey was 36% (this cooperation rate reflects the number of completed interviews [508] divided by the number of eligible individuals reached by telephone [1409]).

### Measures

#### Awareness of changes to screening guidelines

Participants were asked the question “As far as you know, have medical recommendations for the age at which a woman has her first mammogram and how often she should have one changed in the past two years?” Participants were given the response options of yes and no. A don’t know response option was not explicitly offered but was coded if the respondent said don’t know. The “two year” timeframe was chosen to capture the time of the USPSTF recommendation changes (which, depending on the date a given person completed the survey, happened between 12–14 months prior to the survey). To our knowledge, no other US mammography recommendation changes were made in the two year time frame referenced by the question.

#### Accuracy of knowledge about changes to screening guidelines

Those participants who answered yes were then asked an open-ended question about what changes were made to the recommendations. Responses were coded into categories by survey research staff, with codings confirmed by at least one author. Responses were then coded as accurate or inaccurate by the first author. Separate coding was done for accuracy about changes to frequency of screening and changes to age of beginning screening. For changes to frequency, responses were coded as accurate if they indicated a change to every other year or indicated a decrease in frequency without specifying a specific time; other responses (including indicating an increase in frequency, indicating a change to screening every year, and extraneous responses) were coded as inaccurate. Similarly, for the changes to age to begin screening, responses were coded as accurate if they indicated beginning at age 50 or an increase in age without a specific age referent. Other responses were coded as inaccurate.

#### Sources of information about recommendations

Participants who were aware of the USPSTF changes were also asked whether a health care provider had discussed the changes with them and, in an open-ended question, were asked where they heard about the changes. Finally, participants reported how much attention they had paid to reports about the recommendation changes using a 4-point response scale ranging from “no attention” to “a lot of attention”.

#### Attitudes about changes to the screening guidelines

Women who were aware of the changes were asked a single question assessing attitudes about the changes. Attitudes about the changes were assessed on a 4 point response scale with response options of “very good”, “somewhat good”, “somewhat bad”, and “very bad”.

#### Screening advice

All women were asked whether a health care professional had recommended screening to them in the past year.

#### Personal experience with breast cancer and screening

All participants were asked whether they knew anyone who had died from breast cancer, anyone who was currently in treatment or had survived breast cancer, and anyone who had had a false positive result from a mammogram. Participants were asked if they had ever had a mammogram and, if so, when their last mammogram took place.

#### Demographics

Women reported age, health insurance status, whether they had a regular health care provider, race/ethnicity, education level, income level, and state of residence.

#### Analysis strategy

Factors associated with awareness of changes in the USPSTF screening guidelines and with accuracy of knowledge about those changes were examined using logistic regression modeling. Both univariate and multivariate logistic regression equations were estimated with awareness (yes versus no/not sure) as the dichotomous outcome variable and the demographic characteristics hypothesized to be associated with awareness (see *Introduction*; age, race, income, education, knowledge of someone with breast cancer, past screening behavior) as predictor variables; all available demographic variables were included in both the univariate and multivariate models. Because the absolute numbers of respondents from race/ethnic minority groups were relatively low, especially when stratified by age, race was dichotomized to White/non-White for analysis. Because the vast majority of women had a mammogram, the past screening variable was coded as mammogram in last two years (no, yes) for analysis. Analyses were conducted both for the overall sample and stratified by age category (40–49, 50+). Because of concern about possible colinearity between income and education and between the various personal experience variables, we also ran multivariate models including only one of each of these categories of variables. No differences in results were noted.

Sources of knowledge about changes, accuracy of knowledge about recommendations, attitudes about changes, and receipt of medical provider advice about screening were examined with frequency descriptives. The relation of participant age category to each variable was examined with contingency table analysis using Chi-square. A *p*-value of 0.05 was used as the criterion for statistical significance in all analyses.

## Results

### Characteristics of sample

Demographic characteristics of the sample are reported in Table 1. Compared to characteristics of the US population of women over the age of 40 (based on US Census Bureau data [25-28], the sample had a higher proportion of White respondents (88% in our sample versus 78% in US women over 40), higher educational attainment (46.3% have a Bachelor’s degree or higher, relative to 31.1% of the general population), higher income (in our sample, median income is $60,000-80,000/year versus $29,447 or lower for the general population of women over 40), and higher rates of insurance coverage (95.1% in our sample versus 83.7%).

**Table 1:** Characteristics of the sample (N=508)

**Demographic variable**	**Percentage of sample**
**Age**	
*40-49*	48.7%
*50+*	51.3%
***Race***	
*White/Caucasian*	86.8%
*Black/African American*	7.7%
*Hispanic/Latino*	2.4%
*Asian*	1.2.%
*Native American/Alaskan Native*	0.6%
*Don’t Know/Refused*	1.4%
***Education Level***	
*Less than High School Graduate*	6.0%
*High School Graduate*	21.2%
*Some College*	26.5%
*Bachelor’s Degree or Higher*	46.3%
***Income Level***	
*<$35,000*	16.0%
*$35,000 - < $80,000*	40.3%
*$80,000 - < $120,000*	24.0%
*> $120,000*	19.6%
***Census Region***	
*Northeast*	25.0%
*Midwest*	20.6%
*South*	34.8%
*West*	19.6%
***Insurance Status***	
*Uninsured*	4.9%
*Insured*	95.1%
***Have a Health Care Provider***	
*No*	27.5%
*Yes*	72.5%
***Had a Mammogram In the Past 2 Years***	
*No*	16.9%
*Yes*	83.1%

### Awareness of changes to screening guidelines

Results for awareness of changes to the recommendations are summarized in Table 2. Overall, 41.7% of respondents were aware that changes had been made to recommendations. 23.8% of respondents reported that they didn’t know whether changes had been made, with 34.6% reporting that no changes had been made. For further analyses, “don’t know” and “no” responses were collapsed into a single category; given our interest in examining how many women are fully aware of the changes, the comparison of women who are aware (yes responses) to those who are not fully aware is appropriate (note: we also ran analyses with only “yes” and “no” responses included; there were no changes to the patterns of significant results reported here).

**Table 2:** Awareness of changes to USPSTF recommendations (N=508)

	**Yes**	**No**	**Don’t Know**
FULL SAMPLE	41.7%	34.6%	23.8%
Ages 40-49	50.8%	33.1%	16.1%
Ages 50+	33.0%	36.0%	31.0%

Awareness of the change was significantly associated with respondent age; relative to those over age 40–49, women over age4 50 were *less* likely to be aware of the changes in recommendations (for descriptive statistics see Table 2; for odds ratios see Table 3); univariate odds ratio=0.48; multivariate odds ratio controlling for other demographic factors=0.53.

**Table 3:** Factors associated with awareness of changes (N=508)

**Demographic variable**	**Univariate**	**Multivariate**
	**OR (95% CI)**	**OR (95% CI)**
**Age**		
40-49	1.0	1.0
50+	**0.48 (0.33 – 0.68)**	**0.53 (0.34 – 0.85)**
**Race**		
White/Caucasian	1.0	1.0
Non-White/Caucasian	0.67 (0.38 – 1.19)	0.75 (0.39 – 1.44)
**Education Level**		
Less than High School Graduate	1.0	1.0
High School Graduate	2.07 (0.72 – 5.89)	2.60 (0.75 – 8.00)
Some College	**3.75 (1.35 – 10.40)**	3.19 (0.95—10.72)
Bachelor’s Degree or Higher	**4.91 (1.82 – 13.28)**	3.22 (0.998 – 10.56)
**Income Level**		
<$35,000	1.0	1.0
$35,000 - < $80,000	**2.01 (1.04 – 3.91)**	1.41 (0.67 – 3.00)
$80,000 to < $120,000	**2.81 (1.38 – 5.72)**	1.76 (0.77 – 4.04)
> $120,000	**5.08 (2.42 – 10.67)**	**2.87 (1.19 – 6.92)**
**Census Region**		
Northeast	1.0	1.0
Midwest	1.12 (0.66 – 1.88)	1.16 (0.61 – 2.22)
South	0.69 (0.43 – 1.11)	0.79 (0.44 – 1.43)
West	0.85 (0.50 – 1.45)	1.23 (0.64 – 2.37)
**Insurance Status**		
Uninsured	1.0	1.0
Insured	1.28 (0.55 – 2.96)	1.18 (0.40 – 3.46)
**Have a Health Care Provider**		
No	1.0	1.0
Yes	1.04 (0.70 – 1.54)	0.94 (0.56 – 1.60)
**Know Someone Who Died from Breast Cancer**		
No	1.0	1.0
Yes	1.34 (0.93-1.93)	1.10 (0.70-1.72)
**Know Someone Living With/Cured of Breast Cancer**		
No	1.0	1.0
Yes	1.80 (1.11-2.93)	1.26 (0.69-2.27)
**Know Someone with a Mammogram False Positive**		
No	1.0	1.0
Yes	1.51 (0.98-2.33)	1.47 (0.86-2.52)
**Had Mammogram In Past Two Years**		
No	1.0	1.0
Yes	0.74 (0.57-1.19)	0.64 (0.34-1.18)

NOTE: All analyses are logistic regressions with awareness of guidelines changes as a dichotomous outcome variable. Multivariate analysis includes all variables listed in the table as predictors.

### Demographic and personal experience factors associated with awareness of changes

We next examined factors associated with awareness of the changes in recommendations; results are summarized in Table 3. As can be seen in the table, in addition to the age effects reported above, higher income was significantly associated with greater awareness in both univariate and multivariate analyses; in addition higher levels of education and knowing someone living with/cured of breast cancer were both associated with greater awareness in univariate, but not multivariate analyses. In analyses stratified by participant age (results not shown in table; full analyses are available from the first author), the only differences as a function of age were that knowing someone living with breast cancer was associated with increased awareness for those 50 and over but not those 40–49.

### Accuracy of knowledge about changes to screening guidelines

Women who reported being aware of the changes were asked to report what changes had been made. These responses were categorized. The percentage of respondents describing different types of changes are reported in Table 4. As can be seen in the table, for knowledge about changes to start age, 29.7% of respondents provided an accurate answer (i.e., either indicated starting at age 50 or indicated starting later with no specific age mentioned). For knowledge about changes to recommended frequency, 36.1% of respondents provided an accurate answer (i.e., indicated a shift to every other year or indicated a decrease in frequency without a specific time referent).

**Table 4:** Knowledge of changes to mammography recommendations

**Response Category**	**Percentage of Responses**
**Responses About Changes in Age To Start Screening**	
**Accurate Responses About Guidelines Change**	
Start at 50	9.9%
Example: “Used to be 35 or 40 but now it’s 50”	
Start later (no specific age mentioned)	19.8%
Example: “Raised the age”	
**Inaccurate Responses About Guidelines Change**	
Start at age earlier than 50 (specific age mentioned)	6.4%
Example: “Age 45 recommend every two years”	
Start earlier (no specific age mentioned)	10.3%
Example: “changed the age in which to get first mammogram”	
Change in age (no direction mentioned)	4.4%
Example: “changed the age in which to get first mammogram”	
Don’t Know/Uncategorizable	21%
Example: “Insurance changes”, “I don’t know”	
Response did not include statement about age	27.9%
**Responses About Changes to Frequency of Screening**	**Percentage of Responses**
**Accurate Responses About Guidelines Change**	
Every two years	16.8%
Example: “went from every year to two years”	
Less frequent (no specific timetable mentioned)	19.3%
Example: “Length between increase”	
**Inaccurate Responses About Guidelines Change**	
Every year	3.9%
Example: “lower the start age to 35 and made it annually”	
More frequent (no specific timetable mentioned)	8.9%
Example: “I think they want you to have them more often”	
Change in frequency ( no direction mentioned)	4.0%
Example: “The year was changed and how often”	
Don’t Know/Uncategorizable	21%
Example: “More research done”, “I don’t know”	
Response did not include statement about frequency	26.1%

When accuracy for *both* frequency and age were considered together, only 11.9% of respondents reported correctly both age change (e.g., start later or start at age 50) and frequency change (decrease the frequency or every two years). We examined whether accuracy of knowledge (both separate age and frequency accuracy and combined accuracy) was predicted by any demographic or personal experience variables. Accurate knowledge did not vary as a function of any demographic variable.

### Sources of knowledge about changes to screening guidelines

Those women who responded that they were aware of the changes to recommendations were asked where they heard about the changes, how much attention they paid to media reports about the changes, and whether a health care provider had discussed the changes with them. The vast majority of respondents heard about the changes from the news media (80.6%), with health care professionals (4.7%) and friends/family (5.7%) being a much lower source of this information. About one-quarter (26.9%) reported paying a lot of attention to reports about the changes, 42% reported paying some attention, and 29.7% reported paying little or no attention. About one-quarter (25%) reported that a health care provider had talked with them about the changes to recommendations. Reported source of knowledge did not vary as a function of participant age, χ^2^(4)=3.99, *ns.*

### Attitudes about changes to screening guidelines

Finally, those women who were aware of the changes were asked whether they thought the changes were good or bad. 11% of the respondents who were aware of changes reported that the changes were very good, 22% responded that they were good, 33% that they were bad, and 33% that they were very bad. We examined predictors of attitudes about the changes in both univariate and multivariate analyses. The only significant predictor in both univariate and multivariate analyses was participant income, with higher incomes associated with negative attitudes; univariate b=−0.12, *t* (146)=−2.92, *p* < .01; multivariate b=−0.10, *t* (123)=−2.12, *p* < .05. Women who were aware and had accurate knowledge of the changes had more negative attitudes about the changes; accurate about frequency *b* = −0.58, *t* (184)=−3.90, *p* < .001; accurate about age of start *b* = −0.42, *t* (184) = −2.67, *p* < .01.

### Advice about screening

All women were asked whether, in the past year, a health care professional had recommended a mammogram. Overall, about 2/3 of women had received a recommendation (62.6%). The relation of age to mammogram recommendations was examined in both a univariate analysis and in a multivariate analysis controlling for demographic characteristics. Age category was *not* a significant predictor in either analysis; univariate OR=0.74, 95% CI 0.52 – 1.07; multivariate OR=0.65, 95% CI 0.40-1.04. Women under age 50 were no less likely to report having received a mammogram recommendation (66.1% yes) than those over 50 (59.1% yes).

## Discussion

To our knowledge, this is the first examination of women’s awareness of and knowledge about the changes in USPSTF recommendations for breast cancer screening; one recently published report assessed degree of attention paid to changes but did not directly assess whether respondents were aware that changes had been made [22]. Given that the data reported here were collected one year following the release of the USPSTF recommendations, the findings represent an important initial benchmark for women’s knowledge – and gaps in knowledge – of breast cancer screening recommendations, which may anticipate the initial public health impact of these adjustments. Indeed, enough time had passed (14 months) to allow for average-risk women to have developed some knowledge concerning the new recommendations, and to have engaged in potential conversations with their physicians about whether to undergo breast cancer screening. Although a cross-sectional assessment of recommendation awareness must of necessity be conducted at a given time point after the recommendation release, in the context of screening 12–14 months post recommendation is a reasonable time point – it is sufficiently far from the recommendation release to allow individuals to see media coverage, speak with friends and family, or have conversations with a healthcare provider. In the context of screening behavior, health care provider conversations are a known influence on screening [29,30], so setting a data collection timepoint that allows for such conversations is important. In the US, conversations with primary care providers often happen at an annual exam, either by a general practitioner or a gynecologist. Thus, selecting a time point one year after recommendation release increases the likelihood of at least one provider visit having occurred, in addition to allowing time for media exposure and other conversations.

We found levels of awareness of USPSTF recommendations for breast cancer screening in the moderate range, with awareness differing by age group. Half (51%) of women aged 40 to 49 were aware that breast cancer screening recommendations had changed; 33% of women aged 50 and over were aware. Certainly the particular relevance of the recommendations for women in the younger age group may have enhanced the salience of this new information for these women.

The context surrounding the recommendation changes should be noted. The USPSTF is only one of several professional organizations which makes recommendations about screening frequencies. Other prominent professional organizations (e.g., the American Cancer Society) did not change recommendations. As noted in the introduction, there was widespread controversy and publicity about the changes and the disagreements across professional organizations. On the one hand, the differences across professional organizations might account for the low levels of awareness (e.g., if a woman uses another professional organization to look for screening information or is cared for by a provider who follows another organization’s recommendations). On the other hand, the widespread publicity generated because of the context of controversy and disagreement would lead one to expect higher levels of awareness given the media coverage.

Higher education level and higher income were both associated with increased awareness of the recommendation changes in univariate analyses (and for income, also in multivariate analyses). These important socioeconomic predictors of awareness (higher educational attainment and income) are in line with the “knowledge gap hypothesis,” which proposes that information disseminated by mass media is acquired by those at higher socioeconomic status at a faster rate as compared to those of lower socioeconomic status. Given this, that gaps in knowledge acquisition tend to increase over time, and are particularly dramatic in the context of new innovations [31-33]. Differential access to information about cancer prevention, early detection, and treatment options has been proposed as an important factor that perpetuates cancer disparities [34].

This is highly relevant in the context of cancer prevention and control for those at increased risk as well as in the general population, where rapidly burgeoning knowledge of cancer risk factors, prevention strategies, early detection recommendations, and treatment options require concomitant knowledge transfer through multiple communication channels. Uptake of mammography among women in underserved populations, especially among individuals with limited health literacy, has never reached the high rates achieved with more advantaged women [18]. However, it is important to note that this study was not designed nor powered to test the knowledge gap hypothesis.

Despite moderate levels of awareness of the changes in recommendations among these women, their more concrete knowledge of the recommendations was relatively low. We surveyed women concerning their knowledge of how recommendations for breast cancer screening had changed. Only 12% of women surveyed could accurately report both the age and frequency change. For starting age, after “don’t know,” the most frequently endorsed option was “start mammography later,” with 20% endorsing this response. For frequency, after “don’t know,” the most frequently endorsed option was, ‘less frequent,” with 19% endorsing this response. These findings reflect comprehension of the general gist of the new recommendations among sizable minorities of these women, yet low levels of specific knowledge of the new recommendations. This low level of knowledge is consistent with the findings of other work examining responses to the recommendations [22].

It is relatively unsurprising that women’s knowledge of the recommendations is vague considering the fact that women predominantly heard about the new recommendations from the media (81%) rather than from their physicians (5%). These findings highlight the limits of media exposure and an important opportunity for personalized discussion of breast cancer screening benefits and drawbacks in the medical setting. There is an important research priority to prepare healthcare providers to address the challenges of these discussions with their patients. Discussions with women aged 40–50 will shift to an emphasis on the benefits and drawbacks of mammography, based on the preferences of individual patients [35]; discussions with women over age 50 will likely need to cover the rationale for recommended screening every two, rather than one, year. In all cases, these discussions will necessarily go beyond risks and benefits of mammography as they may be emotionally complex, and need to address cognitions and affect about mammography, as well as breast cancer more specifically. The impact of the USPSTF recommendations appears to be relatively slight. We found no differences in whether women received a recommendation to complete a mammogram in the past year – across both age groups, 63% of women received such a recommendation during the time period December 2009 to December 2010.

These sources of knowledge are also interesting in light of the nature of the recommendation process. The USPSTF’s charge is to serve as an expert panel to make recommendations about service effectiveness based on the scientific evidence. The mission of the USPSTF does not extend to public health communication and education to the lay public about guidelines and those guidelines changes. Thus, the USPSTF’s recommendations are disseminated to the public by others, most immediately the mass media.

In our survey only 25% of those who were aware that changes had been made to recommendations reported any discussions with a healthcare provider about the changes to recommendations. Given that the new guidelines encourage individual decision-making in consultation with a health care provider, strategies to increase such mammography discussions would address an important public health need. This will be particularly important for those under the age of 50. One possible explanation for the low levels of provider discussions is that an unknown portion of providers may be basing their mammography advice on recommendations of different professional organizations whose recommendations did not change (e.g., the American Cancer Society [36] or are guided by individual beliefs about mammography effectiveness [37]. Ultimately, the question of health care provider beliefs about the recommendation changes and on which guidelines they base their clinical decisions is an empirical question. Regardless, the very low rate of provider discussion given the large degree of media coverage, as well as the fact that most women reported learning about the changes from media sources, indicates untapped opportunities to educate and encourage healthcare providers about the new recommendations, as well as methods for providers to engage their female patients in discussions that allow the patients to make personally appropriate decisions about whether to undergo breast cancer screening. In fact, such discussions may be increasingly necessary for public health practice across many spheres.

Given the enhanced cultural acceptance of disclosure and discussion of breast cancer diagnosis and treatment over the past 20 years, we also examined whether social network variables related to awareness of the changes in UPSTF breast cancer screening recommendations. Indeed, women who knew someone diagnosed with breast cancer or someone who had received a false positive finding on a mammogram were more aware of the new breast cancer screening recommendations. While our results are cross-sectional, and thus we cannot infer cause and effect, it may be that the salience of breast cancer to these women enhanced their information-seeking regarding breast cancer screening, where they were more impacted by media reports concerning mammography in general. Interestingly, most women surveyed believed that the new breast cancer screening recommendations were ‘bad’ or ‘very bad’ (66%), revealing their important concerns about the recommendations, as well as the media framing of the event, which emphasized the recommendations’ potential negative ramifications. Interestingly, awareness of changes in USPSTF breast cancer screening recommendations was not related to racial/ethnic minority status, whether women had a regular healthcare provider, insurance status, or geographical location. More research is needed with nationally representative samples in order to confirm these findings.

Finally, we found that those women who were aware of the changes in recommendations generally had negative attitudes about those changes, and that attitudes were more negative for those women who had accurate knowledge about the changes. In light of the fact that knowledge of the changes largely came from media sources, this finding may reflect a social amplification effect. Social amplification through media messages can shape the construction of an issue, and ultimately magnify and enlarge the most negative, emotional or threatening elements, resulting in high levels of public concern [38,39]. Our findings likely reflect media framing of the recommendation change as one of rationing care, or reducing younger women’s access to mammography. This framing and the resultant impact on attitudes and feelings concerning mammography may be reflected in women’s discussions with their physicians regarding mammography in the coming years. Physicians may want to prepare themselves for this with both accurate information and tools to help them address potentially high levels of affect regarding mammography screening and breast cancer worries. The relation of accuracy to attitudes may reflect the fact that accurate knowledge of the changes means that women were aware that the changes reduced the frequency and increased the age of screening. Previous work has shown that beliefs about mammography occur most strongly for women impacted by the changes [40]. Women with accurate knowledge knew the impact the changes might reduce their screening frequency, which might account for the more negative attitudes.

### Limitations

There are important limitations of the current research. First, while our sample was diverse on some demographic characteristics, as it was evenly distributed over geographical locations in the United States, and broadly inclusive of different income levels and educational attainment, and representative of different health care provider statuses, the sample was primarily Caucasian and insured, and had higher income and education than the general population. It should be noted that these sample characteristics likely mean that our finding of substantial lack of awareness is an *underestimate* of the true magnitude of the situation – given the relation of income and education to awareness, it is likely that the general population was less aware of the guidelines changes than is represented by our sample. Future work with larger, national probability sampling will be critical to identify more precise estimates of the impact of the USPSTF breast cancer screening recommendations across diverse elements of the United States population.

Second, our relatively low response rate (36%) may limit our ability to generalize to the overall population; it is possible that our respondents were more knowledgeable and/or motivated than the population at large. While response rates to land-line telephone surveys have been much lower in recent years overall [41], recent analyses suggest that low response rates do not bias estimates of population characteristics [42,43]. However, it is conceivable that the sample we collected was more motivated and/or knowledgeable than the general population. If this is the case, it is likely that general population levels of awareness and knowledge of the new breast cancer screening recommendations are likely even lower than what we reported here.

Finally, given that information about innovations tends to diffuse through the population over time [44], it is important to note that the data presented here represent awareness at one time point (one year following the guidelines presentation); presumably surveys conducted longer after the release of new recommendations would show higher rates of awareness.

## Conclusions

National guidelines for preventive health services likely represent an important galvanizing influence on patient inquiries and requests, as well as physician’s discussions with their patients. Accordingly, examination of level of awareness of USPSTF recommendations among women may be an important early indication of potential changes in public health practice. Our findings indicate that women ages 40 and over are, overall, not aware of the changes made to breast cancer screening recommendations despite the wide media coverage the changes received. In addition, the changes in recommendations are not reflected in age differences in physician recommendations for mammography screening. Women who are aware of the changes predominantly view them negatively. Increasing awareness of the guideline changes and addressing attitudes about the changes is critical to ensuring that women consider and discuss the benefits and drawbacks with their physicians, with the goal of ensuring that they are screened for breast cancer at appropriate intervals.

## Competing interests

The authors report no conflicts of interest.

## Authors’ contributions

The two authors contributed equally to the work reported here. Both had responsibility for study conception and design, data interpretation, and manuscript writing and editing. MTK had responsibility for data analysis. Both authors approved the final version of the manuscript. MTK had full access to all the data in the study and takes responsibility for the integrity of the data and the accuracy of the data analyses.

## Pre-publication history

The pre-publication history for this paper can be accessed here:

http://www.biomedcentral.com/1471-2458/12/899/prepub
